# Value-Based Pricing of US Prescription Drugs: Estimated Savings Using Reports From the Institute for Clinical and Economic Review

**DOI:** 10.1001/jamahealthforum.2022.4631

**Published:** 2022-12-09

**Authors:** Kai Yeung, Lisa Bloudek, Yao Ding, Sean D. Sullivan

**Affiliations:** 1 Kaiser Permanente Washington Health Research Institute, Seattle; 2 The Comparative Health Outcomes, Policy, and Economics Institute, School of Pharmacy, University of Washington, Seattle; 3 Agency for Healthcare Research and Quality, Rockville, Maryland

## Abstract

This cross-sectional study estimates how annual US drug spending would change if prices for prescription drugs were set at the value-based price.

## Introduction

The Inflation Reduction Act^1^ allows Medicare to negotiate prices on a limited set of drugs that are not necessarily the highest priced or lowest value. Across payers, interest in lowering drug spending remains high.

The National Academy of Medicine recommends basing drug prices on value, tying prices to the magnitude of benefit to preserve incentives for innovation.^2^ One way to do this is to set prices to achieve a certain cost-effectiveness threshold. Value-based prices (VBPs) are estimated by the Institute for Clinical and Economic Review (ICER), an independent nonprofit that reviews all available evidence of a drug’s clinical effectiveness vs its economic cost to estimate its value. Reports of ICER reviews are increasingly used by US payers in drug price negotiations. The objective of this study was to estimate how annual US drug spending would change if prices for drugs were set to the ICER-reported VBP.

## Methods

This cross-sectional study was approved by the Kaiser Permanente Institutional Review Board. We followed the Strengthening the Reporting of Observational Studies in Epidemiology (STROBE) reporting guidelines.

We obtained VBPs that would achieve cost-effectiveness thresholds of $100 000 and $150 000 per quality-adjusted life-year (QALY) gained (commonly applied thresholds^2^) from all ICER reports from 2015, the first available year, to 2020. We inflated the VBPs to 2020 US dollars using the health care Personal Consumption Expenditures index.^3^ For drugs with multiple VBPs (owing to multiple indications or dosage forms), we calculated an average VBP for each drug, weighted by the percentage of prescriptions for each indication and dosage in the 2017 to 2019 Medical Expenditure Panel Survey (MEPS; details available in the eMethods 1 and 2 in the Supplement). For drugs with insufficient observations in MEPS, we applied an unweighted average (base case) and the highest and lowest drug-specific VBPs (scenario analyses to account for uncertainty).

We linked VBPs to drug-specific observed net prices and total net sales (representing spending by all payers) in 2020 using data from SSR Health. To estimate expected drug spending after applying VBPs, we multiplied total net sales by the ratio of VBP to observed net price—a method that produces conservative estimates because it increases prices and spending for drugs with observed prices below their VBPs, but factors (eg, competition) may legitimately constrain prices for these drugs. In the specific scenario analyses, we did not increase prices and spending for these drugs. We tested changes in spending before and after applying VBPs using 2-tailed Wilcoxon signed-rank tests with a significance level of *P* < .05. Data analyses were performed from November 1, 2019, to July 1, 2020, using STATA, version 15.1 (StataCorp).

## Results

The study sample comprised 73 unique drugs (Table 1), which accounted for $110.4 billion in annual US drug spending (Table 2), approximately one-fifth of total US drug spending in 2020. Eleven unique drugs had multiple ICER-reported VBPs. Most of the drugs (86.3% and 72.6%, respectively) had observed net prices higher than the VBPs at $100 000 per QALY and $150 000 per QALY thresholds.

**Table 1.** Characteristics of Unique Drugs (n = 73)^a^ Comprising the Study Sample

Characteristic	No. (%)
>$100 000/QALY gained	63 (86.3)
>$150 000/QALY gained	53 (72.6)
Unique drug indications	29
Drugs with >1 VBP, No.	11 (15.1)^b^
>1 Indication with a VBP	9 (12.3)
>1 Dosage form with a VBP	3 (4.1)
Therapeutic area	
Autoimmune	31 (42.5)
Cardiometabolic	6 (8.2)
Genetic	11 (15.1)
Oncologic	12 (16.4)
Other	13 (17.8)
Year of ICER evidence report	
2015	1 (1.4)
2016	7 (9.6)
2017	25 (34.3)
2018	23 (31.5)
2019	6 (8.2)
2020	11 (15.1)

Abbreviations: ICER, Institute for Clinical and Economic Review; QALY, quality-adjusted life-year; VBP, value-based price.

^a^ Defined by a unique active ingredient.

^b^ One drug had multiple VBPs because it had multiple indications and dosage forms.

**Table 2.** Annual Spending per Drug and Total for 73 Unique Drugs, by Value-Based Price (VBP) Scenario, 2020

VBP scenario	Spending per drug, median (IQR), US $ millions			*P* value
	Before VBP^a^	After VBP	Change	
Base case				
$100 000/QALY	788 (341 to 1790)	290 (85 to 994)	–373 (–953 to –87)	<.001
$150 000/QALY	788 (341 to 1790)	531 (141 to 1574)	–164 (–600 to 5)	<.001
No price increase				
≤$100 000/QALY	788 (341 to 1790)	290 (82 to 965)	–373 (–953 to –87)	<.001
≤$150 000/QALY	788 (341 to 1790)	531 (134 to 1352)	–186 (–735 to 0)	<.001
**Highest or lowest drug-specific VBP**				
$/QALY				
$100 000/Highest	788 (341 to 1790)	345 (85 to 1073)	–369 (–840 to –87)	<.001
$100 000/Lowest	788 (341 to 1790)	280 (85 to 915)	–373 (–1020 to –87)	<.001
$150 000/Highest	788 (341 to 1790)	546 (141 to 1619)	–153 (–481 to 21)	.005
$150 000/Lowest	788 (341 to 1790)	531 (141 to 1332)	–186 (–747 to 2)	<.001
**Total spending, US $ millions**				
**VBP scenario**	**Before VBP** ^a^	**After VBP**	**Change**	**% Change**
Base case				
$100 000/QALY	110 380	70 071	–40 309	–36.5
$150 000/QALY	110 380	98 604	–11 776	–10.7
No price increases				
≤$100 000/QALY	110 380	52 862	–57 518	–52.1
≤$150 000/QALY	110 380	72 028	–38 351	–34.7
**Highest or lowest drug-specific VBP**				
$/QALY				
$100 000/Highest	110 380	74 303	–36 077	–32.7
$100 000/Lowest	110 380	66 191	–44 189	–40.0
$150 000/Highest	110 380	106 134	–4246	–3.8
$150 000/Lowest	110 380	91 751	–18 629	–16.9

Abbreviation: QALY, quality-adjusted life-year.

^a^ Represents observed spending.

In the base case, applying VBPs at $100 000 per QALY and $150 000 per QALY reduced the median spending per drug by $373 million (IQR, $87 million-$953 million; *P* < .001) and $164 million (IQR, –$5 million to $600 million; *P* < .001). This reduction equates to estimated total annual savings of $11.8 billion (11%) to $40.3 billion (37%) for the 73 drugs. Scenario analyses without price increases produced estimated savings of $38.4 billion (35%) to $57.5 billion (52%).

## Discussion

To put these $11.8 billion to $40.3 billion base case estimates in perspective, total Medicare Part D spending in 2020 was $89 billion.^4^ Alternative approaches (eg, reference pricing) could also achieve savings but may not reflect the value preferences of US populations and could increase prices in the reference country.^5^

A study limitation was that we calculated the total national savings based on average net prices, although prices and savings vary by individual payers. The data from SSR Health included drugs that accounted for more than 90% of US branded drug sales and excluded certain drugs, eg, those marketed by privately held companies.^6^

The findings of this cross-sectional analysis suggest that applying the ICER-reported VBPs to prescription drugs would yield a substantial savings for US health care payers. Both private and public payers have a substantial policy interest in lowering drug prices, and using VBPs may align prices with health benefits.

## Notes

Supplement. **eMethods 1.** Calculating a Single Value-Based Price for Drugs with Multiple Value-Based Prices **eMethods 2.** Calculating Annual Drug Dosages
