# Supplementary material for: Value-Based Pricing of US Prescription Drugs: Estimated Savings Using Reports From the Institute for Clinical and Economic Review
Source: JAMA Health Forum. 2022 Dec 9;3(12):e224631. doi: 10.1001/jamahealthforum.2022.4631 (PMC9856524; doi:10.1001/jamahealthforum.2022.4631)
Supplement: Supplement. — eMethods 1. Calculating a Single Value-Based Price for Drugs with Multiple Value-Based Prices eMethods 2. Calculating Annual Drug Dosages [file jamahealthforum-e224631-s001.pdf]

Supplemental Online Content

Yeung K, Bloudek L, Ding Y, Sullivan SD. Value-based pricing of US prescription drugs: estimated savings using reports from the Institute for Clinical and Economic Review. *JAMA Health Forum.* 2022;3(12):e224631.
doi:10.1001/jamahealthforum.2022.4631

eMethods 1. Calculating a Single Value-Based Price for Drugs with Multiple Value-Based Prices

eMethods 2. Calculating Annual Drug Dosages

This supplemental material has been provided by the authors to give readers additional information about their work.

© 2022 Yeung K et al. *JAMA Health Forum*

eMethods 1. Calculating a Single Value-Based Price for Drugs with Multiple Value-based Prices

For the 11 drugs with multiple Institute for Clinical and Economic Review (ICER)-reported value-based prices (VBPs) (due to multiple indications or dosage forms), we calculated a single VBP for each drug so we could link the drug's ICER-reported VBP data with pricing and sales data (which are reported at the drug level) from SSR Health[1] (a dataset containing drug-level net drug prices and total annual sales). To do this, we applied the following two-step approach.

Step One: Calculate an average VBP for each drug weighted by the percent of prescriptions for each indication and dosage form for that drug

Dr. Ding, a senior economist at the Agency for Healthcare Research and Quality, accessed an unmasked version of the Medical Expenditure Panel Survey (MEPS) prescribed medicines files from 2017 to 2019 to obtain prescription drug fills for the subset of drugs with multiple VBPs. This version of MEPS data does not mask orphan medicines or indications. In contrast, the public version of MEPS masks the identity of orphan drugs used by fewer than 200,000 people.[2] Dr. Ding then linked each prescription fill from the MEPS Prescribed Medicines file to the indication reported by the survey respondent for that particular prescription fill as recorded in the 2017-2019 MEPS Condition file using the Appendix to events file, which links medical conditions with particular events (such prescription fills), as described in a previous study.[3] This resulted in a dataset with counts of prescription drug fills by indication and dosage form. We then applied MEPS survey weights to produce nationally representative counts. Then, for each indication and dosage form for each drug, we calculated the percent of prescriptions that were attributed to a specific indication and dosage form, after excluding indications and dosage forms that do not have an ICER-reported VBP. For example, for the biologic treatment etanercept, ICER reported separate VBPs for two indications: rheumatoid arthritis and plaque psoriasis. However, MEPS respondents reported using etanercept for rheumatoid arthritis, plaque psoriasis, and asthma. To calculate the percentage of prescriptions that were for rheumatoid arthritis and plaque psoriasis, we excluded asthma from the denominator. We then calculated the mean VBP for each drug across indications and dosage forms, weighted by percent of prescription fills. There were 3 drugs matched to MEPS using step one.

Step Two: Conduct scenario analyses for drugs lacking MEPS or ICER data

Drugs were excluded from step one if 1) more than 25% of their use in MEPS was for indications without an ICER-reported VBP, or 2) a drug had an ICER-reported VBP for an indication or dosage form that was not observed in MEPs (e.g., we had an ICER-reported VBP for abatacept intravenous dosage form, but it was not observed in the MEPS prescribed-medicines file. Therefore, we excluded it from step one). For these drugs, we calculated unweighted drug-specific average VBPs (base case) and we conducted scenario analyses applying either the highest drug-specific VBP or the lowest drug-specific VBP in separate analyses. There were 8 drugs in step two.

© 2022 Yeung K et al. *JAMA Health Forum*

eMethods 2. Calculating Annual Drug Dosages

We considered the individual aspects of each drug in our calculations of expected annual drug dosages, to arrive at expected annual prices. For drugs dosed by body weight or body-surface area, we calculated dosages using the average body weight or body-surface area of patients included the ICER reports. For drugs not used continuously (e.g., gene therapy or a fixed number of oncology drug cycles), we derived the dosage and number of administrations from the FDA-approved prescribing information (aka, package insert), or if unavailable, from ICER's stated dosing schedule. For combination drugs that include multiple active ingredients in a single formulation (e.g., a combination tablet), ICER provides a single value-based price for those drugs. For drugs often used in combination with one another (e.g., use of steroid with a monoclonal antibody injection), ICER calculates the value-based price for the main treatment but typically includes the costs and clinical consequences (if substantive) of the co-administered drug in the calculation of the value-based price for the main treatment.

References

1. Hernandez I, San-Juan-Rodriguez A, Good CB, Gellad WF. Changes in list prices, net prices, and discounts for branded drugs in the US, 2007-2018. *JAMA*. Mar 3 2020;323(9):854-862. doi:10.1001/jama.2020.1012
2. Agency for Healthcare Research and Quality. MEPS HC-213A: 2019 Prescribed Medicines: 2.6.2.2 Prescribed Medicine Attributes (RXNAME-RXDAYSUP). *Medical Expenditure Panel Survey*. Department of Health & Human Services; 2021. Accessed June 2, 2022. https://meps.ahrq.gov/data_stats/download_data/pufs/h213a/h213adoc.shtml#Prescribed2622
3. Ding Y, Miller GE, Hill SC. Pre-COVID-19 Retail Use and Expenditures for Drugs That Were Subsequently Used to Treat COVID-19. *Research Findings #49*. Agency for Healthcare Research and Quality; 2021. Accessed May 27, 2022. https://meps.ahrq.gov/data_files/publications/rf49/rf49.shtml

© 2022 Yeung K et al. *JAMA Health Forum*
